# Comparison of detection performance of soft tissue calcifications using artificial intelligence in panoramic radiography

**DOI:** 10.1038/s41598-022-22595-1

**Published:** 2022-11-09

**Authors:** Yool Bin Song, Ho-Gul Jeong, Changgyun Kim, Donghyun Kim, Jaeyeon Kim, Hyung Jun Kim, Wonse Park

**Affiliations:** 1 Department of Advanced General Dentistry, Yonsei University College of Dentistry, 50-1 Yonsei-Ro, Seodaemun-Gu, Seoul, 03722 Republic of Korea; 2 InVisionLab Inc., Seoul, Republic of Korea; 3 CSPA, Korea National Sport University, Seoul, Republic of Korea; 4 Department of Oral and Maxillofacial Surgery, Yonsei University College of Dentistry, Seoul, Republic of Korea

**Keywords:** Panoramic radiography, Dentistry

## Abstract

Artificial intelligence (AI) is limited to teeth and periodontal disease in the dental field, and is used for diagnosis assistance or data analysis, and there has been no research conducted in actual clinical situations. So, we created an environment similar to actual clinical practice and conducted research by selecting three of the soft tissue diseases (carotid artery calcification, lymph node calcification, and sialolith) that are difficult for general dentists to see. Therefore, in this study, the accuracy and reading time are evaluated using panoramic images and AI. A total of 20,000 panoramic images including three diseases were used to develop and train a fast R-CNN model. To compare the performance of the developed model, two oral and maxillofacial radiologists (OMRs) and two general dentists (GDs) read 352 images, excluding the panoramic images used in development for soft tissue calcification diagnosis. On the first visit, the observers read images without AI; on the second visit, the same observers used AI to read the same image. The diagnostic accuracy and specificity for soft tissue calcification of AI were high from 0.727 to 0.926 and from 0.171 to 1.000, whereas the sensitivity for lymph node calcification and sialolith were low at 0.250 and 0.188, respectively. The reading time of AI increased in the GD group (619 to 1049) and decreased in the OMR group (1347 to 1372). In addition, reading scores increased in both groups (GD from 11.4 to 39.8 and OMR from 3.4 to 10.8). Using AI, although the detection sensitivity of sialolith and lymph node calcification was lower than that of carotid artery calcification, the total reading time of the OMR specialists was reduced and the GDs reading accuracy was improved. The AI used in this study helped to improve the diagnostic accuracy of the GD group, who were not familiar with the soft tissue calcification diagnosis, but more data sets are needed to improve the detection performance of the two diseases with low sensitivity of AI.

## Introduction

Panoramic images are widely used in dentistry to screen for general pathological features in the maxillofacial area because they show a wide range of areas with only minimal radiation exposure and at low-cost^1,2^. Many diseases, including soft tissue calcification, can be diagnosed through panoramic images.

A soft tissue calcification in the facial area is uncommon; however, radiographic diagnosis often matches the final diagnosis^3^. The most important diagnostic criteria are anatomical location; distribution; and the number, size, and shape of calcifications^4^. Panoramic imaging can reveal typical soft tissue calcifications, such as carotid artery calcification, lymph node calcification, and sialolith. These diseases are likely to be missed if general dentists (GDs) only diagnose the pathological condition of the teeth and surrounding tissues in the images.

Sialolith is a disease that occurs in the parenchyma or duct of salivary glands, primarily in the submandibular or parotid glands^5^, and most sialolith instances observed in panoramic images occur in the submandibular gland. Sialolith is often accompanied by clinical symptoms, such as intermittent pain or swelling of the affected salivary gland^6^; therefore, even GDs who have not undergone professional image reading training are more likely to diagnose it using panoramic images than diagnosing other soft tissue calcifications similarly.

However, lymph node or carotid artery calcifications have no clinical symptoms; therefore, if a general dentist does not observe a panoramic image closely, they might be missed. A lymph node calcification indicates the possibility of chronic systemic infections such as tuberculosis, and because most of them have no clinical symptoms, they are often accidentally diagnosed through panoramic images. Additionally, most of them appear unilateral, and in the oral and maxillofacial areas, they are often observed below or behind the mandibular angle, which is close to the locations where sialolith of the submandibular glands occurs^7^. In a panoramic image, sialolith is often observed as a single round radiopacity, and a lymph node calcification is often observed as multiple irregular radiopacities; therefore, it is possible to tell them apart, although in some cases, it might be difficult to differentiate between the two.

A carotid artery calcification is caused by blockage of arteries due to the gradual accumulation of calcified plaques^8^ and can cause ischemic cerebrovascular insults due to severe stenosis and occlusion. Moreover, carotid artery disease may not show symptoms until severe contraction or complete blockage of the carotid artery occurs, which may indicate the possibility of other cardiovascular or cerebrovascular diseases. Most of such calcifications do not have specific clinical symptoms, but they may be still dangerous and result in sudden death if appropriate measures are not taken^8^.

Therefore, an accurate early diagnosis of carotid artery disease can be of significant help to patients^9^. In panoramic images, carotid artery calcifications are generally characterized by linear irregular radiopaque features around the hyoid bone and the third and fourth cervical vertebras^8,10^. Because carotid artery calcification can be an indicator of other serious diseases, an accurate diagnosis is crucial for dentists to determine a course of treatment^8^.

Recently, artificial intelligence (AI) has been widely used for aiding, E-learning, data analysis in the diagnosis in the medical and dental imaging fields^11–13^. In the field of dental imaging, AI-assisted diagnosis is mostly limited to dental and periodontal diseases, but AI has recently been developed and applied towards diagnosing various specific features, including soft tissue calcification^14^ and tissue tumors^15^. In addition, Chane-Vese model for unsupervised learning is used for medical image segmentation^16^.

However, there are few studies on the accuracy of AI-assisted imaging diagnosis in the dental field in an actual clinical environment. In addition, there is limited evaluation of the effectiveness of AI-assisted imaging as a diagnostic aid tool for dentists, compared to the existing diagnostic methods.

In this study, we compared and evaluated the effects of deep-learning-based AI algorithms, when used by oral and maxillofacial radiologists(OMR) as well as GD on the accuracy and time required for diagnosing soft tissue calcifications (carotid artery calcification, lymph node calcification, sialolith) or normal features.

## Materials and methods

### Datasets

This study was approved by the Institutional Review Board of Yonsei University Dental Hospital (IRB No. 2-2021-0024). De-identified participant data were used in this retrospective study and therefore, the written consent requirement was waived. This study was performed in accordance with the Declaration of Helsinki. The criteria for selection were panoramic images of patients diagnosed with sialolith and lymph node calcification after visiting the Department of Advanced General Dentistry and Department of Oral and Maxillofacial Surgery at Yonsei University Dental Hospital from June 2006 to November 2020. Moreover, among the patients admitted to the departments of Cardiovascular Surgery or Cardiology of Yonsei University Severance Hospital and requested collaboration with the Department of Integrated Dentistry at Yonsei University Dental Hospital from June 2006 to November 2020, those diagnosed with carotid artery calcification on panoramic images were used.

Among 163 patients diagnosed with sialolith, patients with panoramic radiographs were primarily screened. Afterwards, 60 patients were randomly selected to be used for AI testing. There were 26 patients who were diagnosed with lymph node calcification and took panoramic radiographs were all selected. For carotid artery calcification, 3928 patients with panoramic radiographs were first screened, and then 60 of them were randomly selected.

Regarding the exclusion criteria, cases with only a preliminary/presumed diagnosis from the medical record or panoramic image but without a definitive diagnosis were excluded. Cases with medical records but no panoramic images, or when soft tissue calcification was not clearly observed in the study, were also excluded (Table 1).

**Table 1:** Characteristics description of the datasets.

Category	Location	1st reading	2nd reading
Carotid artery calcification	Both	19	17
	Right	4	3
	Left	7	10
Lymph node calcification	Both	9	9
	Right	7	7
	Left	10	10
Sialolith	Right	12	17
	Left	18	13
Normal		90	90
Total		176	176

### Fast region-based convergence neural network system

In this study, rather than complex processes and pretreatments, a fast region-based convergence neural network (FAST-RCNN) with ResNet Backbone that detected suspected sialolith, lymph node calcification, and carotid artery calcification was used to assist GDs as well as OMRs in diagnosing the three diseases through panoramic images. ResNet improves the accuracy by reducing the depth of the learning layer and increasing the performance compared of the convolutional neural network (CNN) model, which is an existing image analysis model, through residual learning. Therefore, Fast-RCNN divides the characteristics of objects in sialolith, lymph node calcification, and carotid artery calcification, into CNN-based feature maps with different characteristics and then trains them through a CNN. Subsequently, the feature map from the CNN passes through a region proposal network that evaluates the degree of detection judgment^17^, receives the classification and interval values of the object, and resizes the box to be placed in the fully connected (FC) layer using ROI pooling.

Fast-RCNN is a method that derives better accuracy than existing object detection algorithms by extracting image features and minimizing noise for image analysis. Fast-RCNN is composed of a convolution feature map and ROI feature vector. The convolution feature map delivers images to the convolution and max-pooling layers, and the received information is placed as features in the ROI feature vector map. Now, we can apply classification and bounding box regression to this vector to obtain each loss, and train the entire model by back propagating it. At this time, it is necessary to properly weave the classification loss and the bounding box regression, which is called a multi-task loss. The formula is as follows (1). First, as an input, p is the probability value of K + 1 (K objects + 1 background, class representing no object) obtained through soft max. where u is the ground truth label value of the ROI. Next, we apply bounding box regression to the result, which returns t_k_ values that adjust the x, y, w, and h values for K + 1 classes, respectively. In the loss function, only the value corresponding to the ground truth label among these values is fetched, which corresponds to t_u_. The v corresponds to the ground truth bounding box adjustment value.

1 $$\begin{array}{*{20}l} {\text{L}}\left( {{\text{p}},{\text{u}},{\text{t}}^{{\text{u}}} ,{\text{v}}} \right) = L_{cls} \left( {p,u} \right) + \lambda \left[ {u \ge 1} \right]L_{loc} \left( {t^{u} , v} \right), \hfill \\ {\text{p}} = \left( {{\text{p}}_{0} , \ldots ,{\text{ p}}_{{\text{K}}} } \right). \hfill \\ \end{array}$$

Then, it receives the bounding box regression prediction value corresponding to the correct answer label and the ground truth adjustment value. For each of x, y, w, and h, the difference between the predicted value and the label value is calculated, and the sum passed through a function called smooth_L1_ is calculated. As a result, the prediction process is completed as (2).

2 $${\text{Smooth}}_{\text{L}1}\left(x\right)= \left\{\begin{array}{ll}0.5{x}^{2}& \text{if }\left|x\right|<1\\ \left|x\right|-0.5& otherwise\end{array}.\right.$$

**Figure 1:**
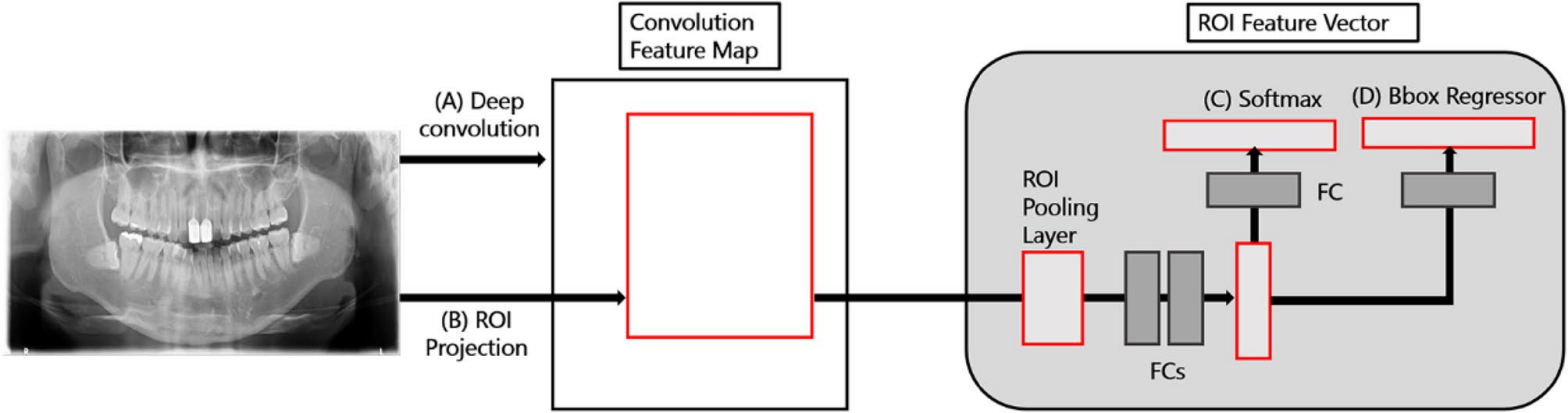
Fast RCNN network model for predicting soft tissue calcification. (A) Deep convolution suggested candidate. (B) Candidate boxes are executed in Pooling Layer. (C) Softmax determines class name of disease. (D) Regressor estimates probability index for each ROI Layer.

This model has a simpler pre-processing and learning process than an algorithm that segments the entire detailed area; additionally, it extracts image features and minimizes noise. Therefore, it showcases a higher accuracy than conventional CNNs^18^. As shown in Fig. 1 Fast-RCNN consists of a convolution feature map (CNN-based) and a region of interest (ROI) derived from propositional feature vectors. The convolution feature map extracts features of an entire image using convolution and max-pooling layers, generates vector values for these features, and delivers them to the ROI pooling layer^19^. Subsequently, the ROI feature vector sets the various ranges of spaces for the features of the received image and converts these features into a map. The converted maps are then moved to the fully connected FC layers. The final image class is determined by calculating the probability for one of the K object classes and then evaluating the same of each set for the K classes. The model was trained for 100,000 epochs, and the learning rate was set from 0.001 through 0.000001. For actual learning, Tensorflow object detection library was used, and training was performed until reaching the maximum step with height and wide strides of 16 for 4 classes.

The Fast-RCNN divides the characteristics of objects in sialolith, lymph node calcification, and carotid artery calcification into CNN-based feature maps with different characteristics and then trains them through the CNN. Subsequently, the feature map from the CNN passes through a region proposal network that evaluates the degree of detection judgment^17^, receives the classification and interval values of the object, and resizes the box to be placed in the FC layer using ROI pooling. This model determined diseases by dividing the cases into a total of four classes, namely sialolith, lymph node calcification, carotid artery calcification, and normal, using the output and loss of values that passed the FC layer to find the optimal category for the object.

### Observer study

**Figure 2:**
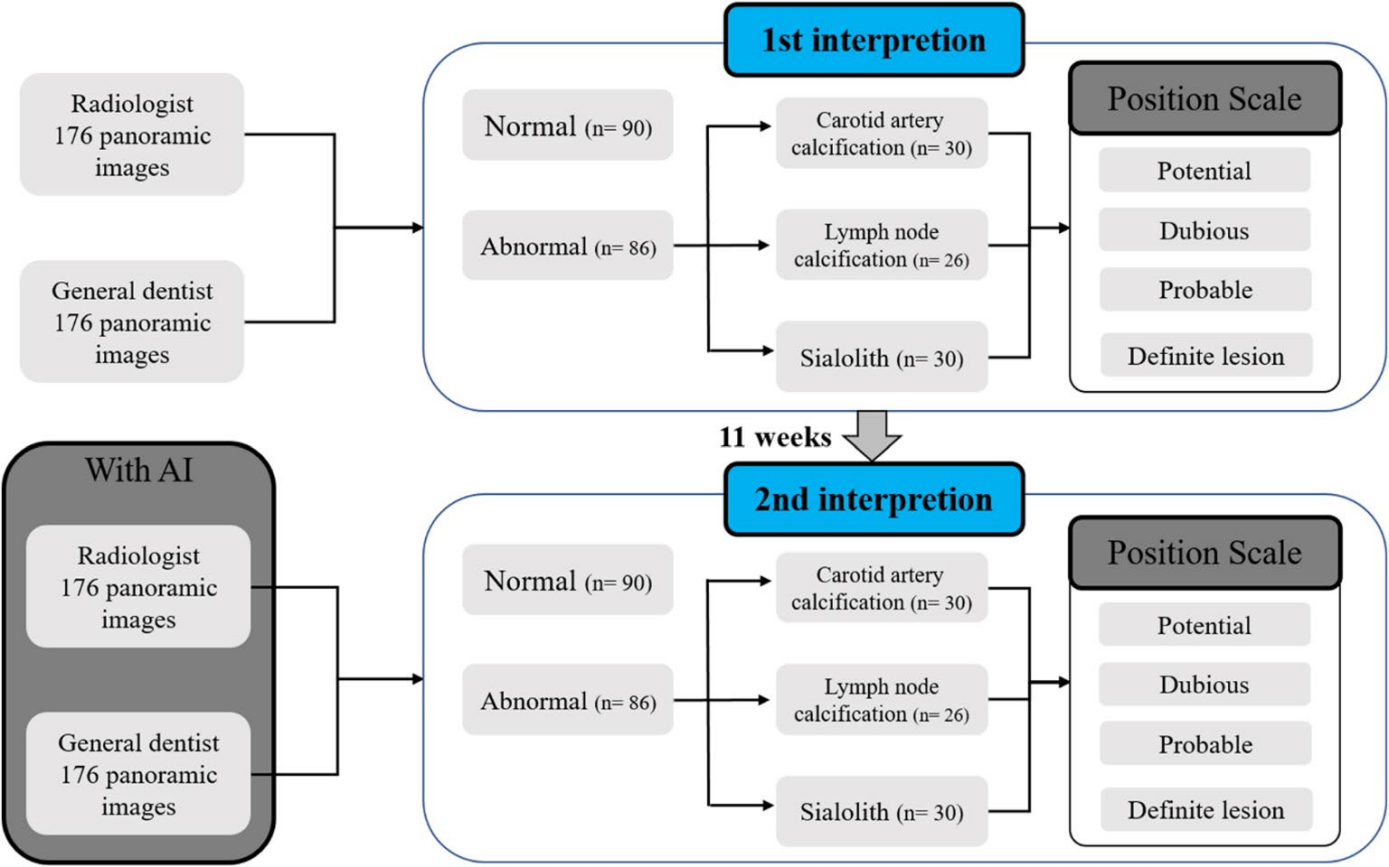
Schematic of observer study design. The first readings were 176 images without artificial intelligence. Abnormal images are separated first and classified as carotid artery calcification, lymph node calcification, or sialolith. Reading confidence is then expressed on a 1 through 4 confidence scale. The second reading takes place 11 weeks after the first reading, and 176 images, different from the first reading, are read using the AI algorithm. Normal and abnormal images are classified first and then a confidence scale of 1 through 4 is displayed.

All observers assessed the presence or absence of sialolith, lymph node calcification, and carotid artery calcification in panoramic images over two sessions on different days, distinguishing left and right (Fig. 2).

In the first reading without AI, each participant watched 176 panoramic images and classified them as normal or abnormal without the help of AI, and if an image was deemed abnormal, they reported the possibility of sialolith, lymph node calcification, and carotid artery calcification on a confidence scale (1–4 points) for left and right side. In the confidence scale, the numbers one, two, three, and four meant potential, dubious, probable, and definite lesions, respectively.

The second reading with AI was conducted 11 weeks after the first reading session. As the panoramic images diagnosed with lymph node calcification were the same as those in the first reading, secondary readings were performed at a sufficient interval to erase the memories of the images. The same observers read under identical conditions, referring to the diagnostic results of the developed AI.

Desktop monitors (HP P24h G4 FHD, screen resolution 1920 × 1080 pixels) were used for reading, and the reading environment around the monitor was the same for both sessions. The time required for the first and second readings of all 176 images together was measured in seconds; no upper limit was specified.

### Data analysis

For each observer, the time required for the first and second readings was compared and evaluated. For each panoramic image, the actual diagnosis and the observer’s diagnosis were compared for both left and right sides—one point was scored if both sides were correct, and zero points if either side was wrong. The perfect score was 176 points per person during each session, which was converted to a scale of 0 to 100 points.

Additionally, the sensitivity and specificity of the observers’ first and second readings were compared and evaluated, and the receiver operating characteristic (ROC) was calculated using scale values. Finally, the accuracy of the AI algorithm was compared and evaluated using the sensitivity and specificity results of the readings.

## Results

### Comparison of reading times and scores using AI

The first reading time of the radiologist group was longer than that of the general dentist group. The time required for the AI-assisted second reading was reduced from the first reading for the radiologist group and increased for the general dentist group (Table 2).

**Table 2:** Individual outcome of observer study without and with AI.

Observer	1st (without AI)	2nd (with AI)
**Time(s)**		
Oral and Maxillofacial Radiologist A	5210	3863
Oral and Maxillofacial Radiologist B	4557	3185
General Dentist A	2248	3297
General Dentist B	3285	3904
**Score**^**1,2**^		
Oral and Maxillofacial Radiologist A	75.0	85.8
Oral and Maxillofacial Radiologist B	67.6	71.0
General Dentist A	27.8	67.6
General Dentist B	53.4	64.8

^1^One point for correct answers (both left and right side) and zero point for incorrect answers (either left or right side).

^2^Converted from a total of 176 points to 100 points.

The first reading score on a scale of 0 to 176 points was 132 and 119 points for radiologists A and B, respectively, and 49 and 94 points for GD A and B, respectively. In the AI-assisted second reading, radiologists A and B scored 151 and 125 points, respectively, and GD A and B scored 119 and 114 points, respectively. In the GD group, the score of A increased significantly when AI was used moreover, in the OMR group, scores for both A and B slightly increased, but there was no significant difference.

### Evaluation of observer performance with and without AI

**Figure 3:**
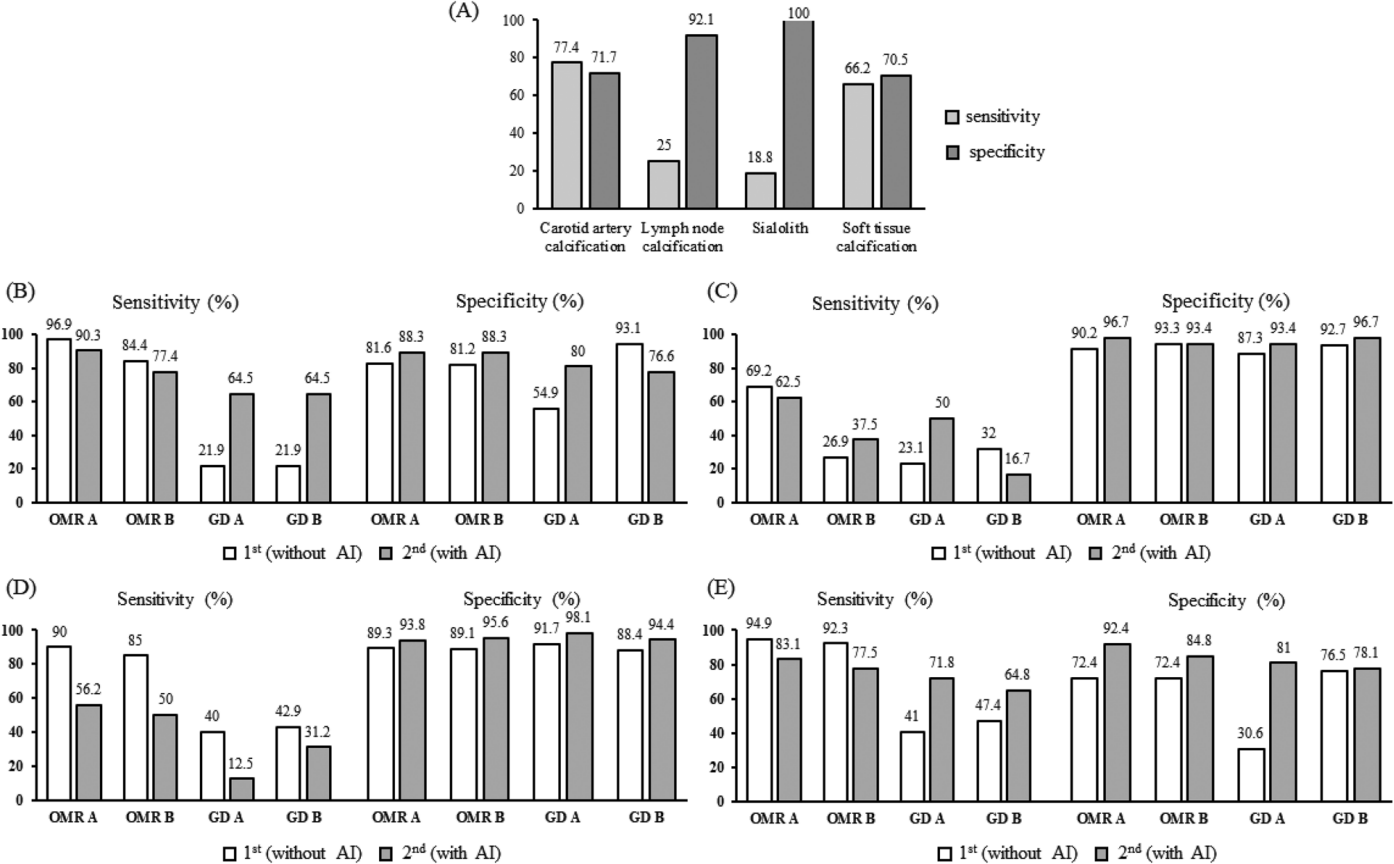
Comparison of AI and observer performances. Sensitivity and specificity of (**A**) artificial intelligence detection for all cases, (**B**) carotid artery calcification, (**C**) lymph node calcification, (**D**) sialolith, and (**E**) all soft tissue calcification with and without artificial intelligence.

**Figure 4:**
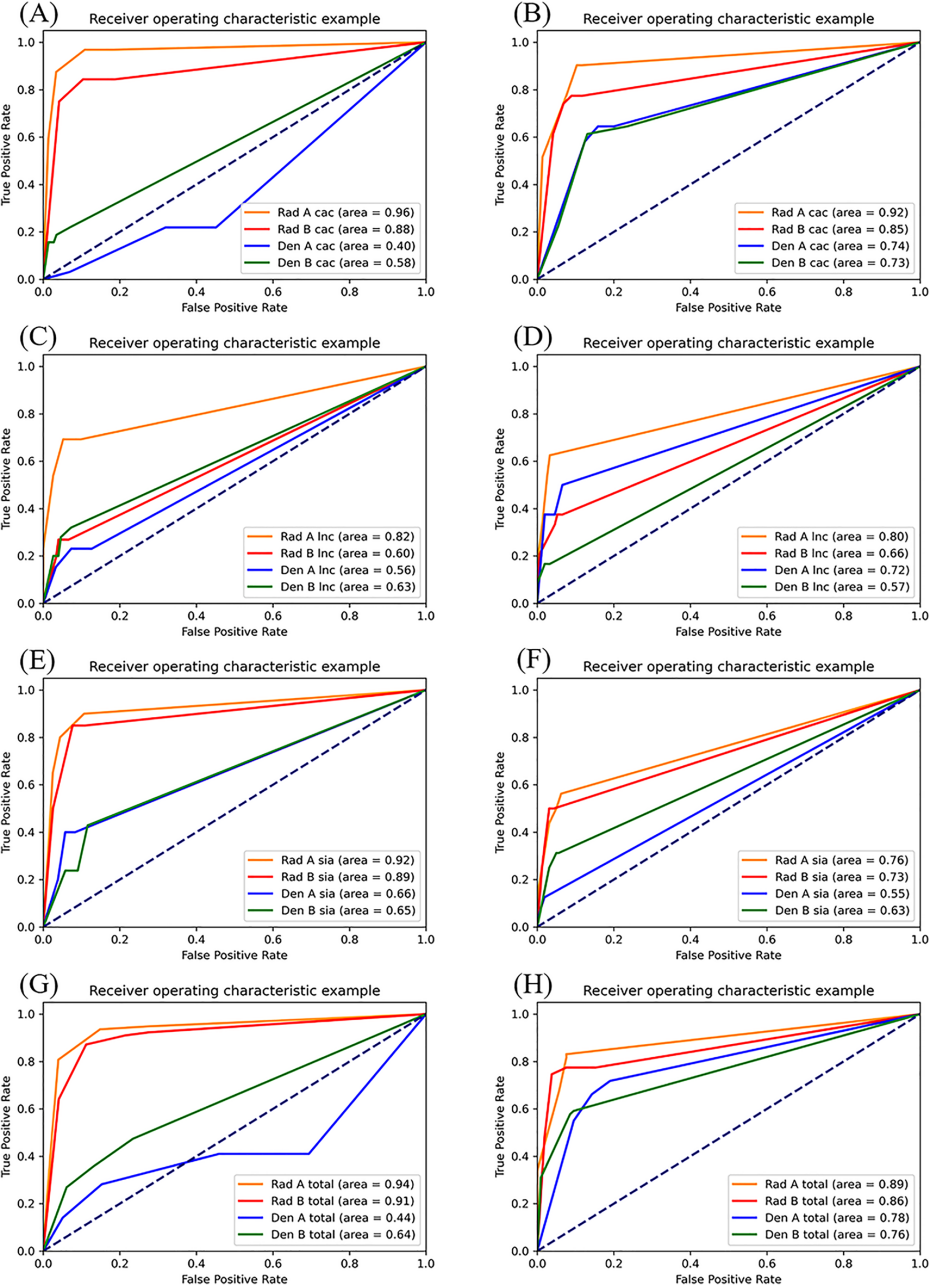
Comparison of carotid artery calcification, lymph node calcification, and sialolith. The comparison of first (**A**) and second (**B**) readings of carotid calcification. The comparison of first (**C**) and second (**D**) readings of lymph node calcification. The comparison of first (**E**) and second (**F**) readings of sialolith. Overall comparison of the first (**G**) and second (**H**) readings of soft tissue calcification.

Comparing the first and second readings in the radiologist group, sensitivity was slightly lower in the second reading than in the first, but specificity was higher in the second (Fig. 3, Table 3). In the GD group, both sensitivity and specificity increased overall, except for the sensitivity for sialolith and lymph node calcification. In the OMR group, sensitivity decreased in all soft tissue calcification, but specificity increased. There was no significant difference between primary and secondary studies in accurately diagnosing carotid artery calcification. However, in the GD group the accuracy improved on the AI-assisted second reading (Fig. 4).

**Table 3:** Sensitivity and specificity results.

Reader			Carotid artery calcification	Lymph node calcification	Sialolith	Soft tissue calcification
Oral and Maxillofacial Radiologist A	Sensitivity	1st	0.969	0.692	0.900	0.949
		2nd	0.903	0.625	0.562	0.831
	Specificity	1st	0.816	0.902	0.893	0.724
		2nd	0.883	0.967	0.938	0.924
	Accuracy	1st	0.841	0.869	0.892	0.818
		2nd	0.886	0.920	0.903	0.886
Oral and Maxillofacial Radiologist B	Sensitivity	1st	0.844	0.269	0.850	0.923
		2nd	0.774	0.375	0.500	0.775
	Specificity	1st	0.812	0.933	0.891	0.724
		2nd	0.883	0.934	0.956	0.848
	Accuracy	1st	0.818	0.835	0.886	0.812
		2nd	0.864	0.858	0.915	0.818
General Dentist A	Sensitivity	1st	0.219	0.231	0.400	0.410
		2nd	0.645	0.500	0.125	0.718
	Specificity	1st	0.549	0.873	0.917	0.306
		2nd	0.800	0.934	0.981	0.810
	Accuracy	1st	0.489	0.778	0.858	0.352
		2nd	0.773	0.875	0.903	0.773
General Dentist B	Sensitivity	1st	0.219	0.320	0.429	0.474
		2nd	0.645	0.167	0.312	0.648
	Specificity	1st	0.931	0.927	0.884	0.765
		2nd	0.766	0.967	0.944	0.781
	Accuracy	1st	0.801	0.841	0.830	0.636
		2nd	0.744	0.858	0.886	0.727
Artificial intelligence	Sensitivity		0.774	0.250	0.188	0.662
	Specificity		0.717	0.921	1.000	0.705
	Accuracy		0.727	0.830	0.926	0.688

As a result of comparative evaluation of the diagnostic accuracy of carotid artery calcification, lymph node calcification, and sialolith, there was no significant difference in the diagnostic accuracy of the primary and secondary readings by the OMR group, but the GD group showed improved diagnostic accuracy.

## Discussion

We analyzed the accuracy of artificial intelligence in detecting soft tissue calcification in panoramic radiographs, and the reading time and correct rate of experts and non-experts to see how this system can help clinically. The sensitivity of the AI system was high for carotid artery calcification but low for sialolith and lymph node calcification, and the readout time increased in GD with AI and decreased in the OMR specialist group. When AI was used, the percentage of correct answers increased.

Several studies have reported that artificial intelligence helps in image reading, and that it helps clinicians^20,21^. In the field of dentistry, artificial intelligence research using panoramic photos has been actively conducted recently; most studies have been on tooth segmentation and tooth number matching^22–24^, detection of primary teeth^25^, and detection of taurodontism^26^, which is a tooth anomaly. Studies on artificial intelligence detection of osteoporosis in panoramic photos are also being actively conducted^27–29^. Studies evaluating the detection of the inferior alveolar neural tube and the relationship between the wisdom tooth and the inferior alveolar canal have also been reported^30–32^ Studies to detect morphological abnormalities such as c shaped root in mandibular second molar have also been reported^33,34^. However, to the best of our knowledge, our research is the first attempt to interpret the shape abnormalities of soft tissues in the head and neck with artificial intelligence.

Detecting calcified findings on panoramic radiography has important clinical significance. In particular, in the case of carotid artery calcification, it is important for cardiovascular disease and stroke prevention^35–37^, and lymph node calcification is known to be mainly related to tuberculosis. Although it is difficult to read easily, it is very important that if you accidentally discover that there is calcification in the head and neck, it can be an important clue to find out the patient’s medical history.

We focused on the three classes of calcification, namely carotid artery calcification, sialolith, and lymph node calcification, to observe the effect of the AI algorithm on the image reading of general dentists. As expected, with the help of AI, the frequency of correcting calcification in GD and OMR increased, but reading time increased in GD and decreased in OMR.

It may appear that reading times were increased with AI so this may be seen as a drawback for clinical practice. But using AI, where the reading time increased within the General Dentistry group, it decreased in the Oral and Maxillofacial Radiology group, so the reading time became similar in both groups. We believe that general dentists might tend to interpret images roughly because of ignorance of calcification, whereas with the help of AI they could dedicate more time and attention to detect abnormal findings.

The AI algorithm used in our study had low sensitivity in sialolith and lymph node calcification; however, its detection performance of carotid artery calcification was satisfactory. This may have resulted from an insufficient amount of data of sialolith and lymph node calcification images. Detection of carotid artery calcification with AI may be beneficial to general dentists. If a patient who visits the dentist is unaware of the seriousness of his or her heart disease, the patient's general medical history may be missed at the interview. If AI can detect carotid calcification in panoramic radiographs, it can identify patients with asymptomatic heart disease. If more cases are collected through multicenter studies, it is expected that the accuracy of sialolith and lymph node calcification detection would be improved.

When comparing the performance of image reading between GDs and OMRs using ROC, the accuracy of diagnosis of carotid artery calcification with AI support decreased slightly in the OMR group. Both ROC curve readings were about 0.9 but the sialolith reading was relatively less accurate. These results should not be compared absolutely because the type and diagnosis of the image were not the same. Earlier studies reported that AI assistance in conventional chest X-ray diagnosis was helpful^38,39^. Therefore, it is worth hypothesizing that the premature prognosis of AI improving the diagnostic accuracy for all diseases by a group of skilled specialists may be incorrect. However, in the GD group, the diagnostic accuracy of carotid artery calcification significantly improved after AI-assistance, which may have affected the overall diagnostic accuracy of carotid artery calcification. The diagnostic accuracy of lymph node calcification improved for general dentist A (see Table 3); however, there was no significant difference among the remaining three. There was little difference in the accuracy of diagnosing the site of soft tissue calcification by the OMR group before and after AI-assistance, but the diagnostic accuracy improved for the GD group. Thus, we propose that AI can improve the diagnostic accuracy of GDs without expertise in diagnosing soft tissue calcifications, and it is particularly effective in diagnosing carotid artery calcification.

These results were also related to the performance of the AI. The diagnostic accuracy of the AI itself was verified using sensitivity and specificity criteria by evaluating the presence or absence of a disease only^40^. The sensitivity for carotid artery calcification was significantly higher than for lymph node calcification and sialolith, and the specificity for lymph node calcification and sialolith was higher than that for carotid artery calcification.

The sensitivity and specificity for carotid artery calcification were 0.77 and 0.71, respectively, and although these were slightly lower than the sensitivity (0.81) and specificity (0.83) of AI-diagnosed dental caries in another study^41^, the results were somewhat similar. The sensitivity of the AI for carotid artery calcification was higher than that of the general dentist group, which might have contributed to the improved diagnostic accuracy of carotid artery calcification by GDs.

Our study has some limitations. First, the main drawback of our study is lower performance regarding detecting sialolith and lymph node calcification. Increasing the number of cases may result in better accuracy of AI, thus, if more cases are collected through multicenter studies, it is expected that the accuracy of sialolith and lymph node calcification detection will be improved. Second, our data is collected in one institution; to evaluate AI performance, multicenter study, and validation are mandatory. Third, there is an imbalance in the number of cases; sialolith and lymph node calcification are rare compared to carotid artery calcification. Our institution is one of the biggest dental hospitals in South Korea, however, we could collect only 163 cases for sialolith and 26 cases for lymph node calcification in 15 years. Lymph node calcification is frequently observed in patients with a history of tuberculosis, but the incidence of tuberculosis in Korea has steadily decreased so far. And, in order to increase the detection performance in carotid artery calcification, we can use more cases of carotid artery calcification. But in this study, we tried to compare the detection performance among three calcifications so an experimental design showing even distribution of three calcification case is more important. So, even though we collected many cases of carotid artery calcification, we had to use only 60 cases of X-ray at this time by random sampling. Fourth, only two GD and OMR specialists tested the detection performance. Research involving more GDs and OMR specialists would lead to more reliable results. Finally, although the AI algorithm we used in this study has been widely used, it is not an up-to-date method. Recently, deep learning algorithms such as zero shot detection, few shot detection, and vector component analysis show relatively high accuracy even with a small amount of data. If we apply those models into our study in the future, improved performance even for a relatively small number of diseases would be possible.

## Conclusions

The AI used in this study helped improve the diagnostic accuracy of GD groups who were not familiar with diagnosing soft tissue calcification. Especially, in carotid artery calcification, AI significantly improved the diagnostic accuracy of GDs. These results indicate that, if utilized well, panoramic imaging can be a useful screening tool to diagnose other diseases.

## Data Availability

The datasets generated during and/or analyzed during the current study are available from the corresponding author on reasonable request.

## References

[1] Maia PRL, Tomaz AFG, Maia EFT, Lima KC, Oliveira PT (2021). Prevalence of soft tissue calcifications in panoramic radiographs of the maxillofacial region of older adults. Gerodontology..

[2] Kim JH (2016). Comparison of the diagnostic performance of panoramic and occlusal radiographs in detecting submandibular sialoliths. Imaging Sci. Dent..

[3] Moreira-Souza L (2019). Brightness and contrast adjustments influence the radiographic detection of soft tissue calcification. Oral Dis..

[4] Garay I, Netto HD, Olate S (2014). Soft tissue calcified in mandibular angle area observed by means of panoramic radiography. Int. J. Clin. Exp. Med..

[5] Sobrino-Guijarro B, Cascarini L, Lingam RK (2013). Advances in imaging of obstructed salivary glands can improve diagnostic outcomes. Oral Maxillofac. Surg..

[6] Jadu FM, Lam EW (2013). A comparative study of the diagnostic capabilities of 2D plain radiograph and 3D cone beam CT sialography. Dentomaxillofac. Radiol..

[7] Kumar GA, Deora SS (2020). Dystrophic calcification in the oral cavity resulting in mechanical dysphagia: A case report and review of calcification in the head and neck region. Cureus.

[8] Nasseh I, Aoun G (2018). Carotid artery calcification: A digital panoramic-based study. Diseases.

[9] Ertas ET, Sisman Y (2011). Detection of incidental carotid artery calcifications during dental examinations: Panoramic radiography as an important aid in dentistry. Oral Surg. Oral Med. Oral Pathol. Oral Radiol. Endod..

[10] Carter LC (2000). Discrimination between calcified triticeous cartilage and calcified carotid atheroma on panoramic radiography. Oral Surg. Oral Med. Oral Pathol. Oral Radiol. Endod..

[11] Moons KG (2012). Risk prediction models: II. External validation, model updating, and impact assessment. Heart.

[12] Agarwal A, Sharma S, Kumar V, Kaur M (2021). Effect of E-learning on public health and environment during COVID-19 lockdown. Big Data Min. Anal..

[13] Wang X, Zhou Y, Zhao C (2021). Heart-rate analysis of healthy and insomnia groups with detrended fractal dimension feature in edge. Tsinghua Sci. Technol..

[14] Khanagar SB (2021). Developments, application, and performance of artificial intelligence in dentistry—A systematic review. J. Dent. Sci..

[15] Tekouabou SCK, Hartini S, Rustam Z, Silkan H, Agoujil S (2021). Improvement in automated diagnosis of soft tissues tumors using machine learning. Big Data Min. Anal..

[16] Huang Q (2021). A Chan-Vese model based on the Markov chain for unsupervised medical image segmentation. Tsinghua Sci. Technol..

[17] Certa A, Enea M, Galante GM, La Fata CM (2017). ELECTRE TRI-based approach to the failure modes classification on the basis of risk parameters: An alternative to the risk priority number. Comput. Ind. Eng..

[18] Kim P (2017). MATLAB Deep Learning.

[19] Yan, K., Huang, S., Song, Y., Liu, W. & Fan, N. *2017 36th Chinese Control Conference (CCC)*, 4077–4081 (IEEE).

[20] Topol EJ (2019). High-performance medicine: The convergence of human and artificial intelligence. Nat. Med..

[21] Shan T, Tay F, Gu L (2021). Application of artificial intelligence in dentistry. J. Dent. Res..

[22] Bilgir E (2021). An artifıcial intelligence approach to automatic tooth detection and numbering in panoramic radiographs. BMC Med. Imaging.

[23] Leite AF (2021). Artificial intelligence-driven novel tool for tooth detection and segmentation on panoramic radiographs. Clin. Oral Investig..

[24] Tuzoff DV (2019). Tooth detection and numbering in panoramic radiographs using convolutional neural networks. Dentomaxillofac. Radiol..

[25] Kılıc MC (2021). Artificial intelligence system for automatic deciduous tooth detection and numbering in panoramic radiographs. Dentomaxillofac. Radiol..

[26] Duman S (2022). Detecting the presence of taurodont teeth on panoramic radiographs using a deep learning-based convolutional neural network algorithm. Oral Radiol..

[27] Nakamoto T, Taguchi A, Kakimoto N (2022). Osteoporosis screening support system from panoramic radiographs using deep learning by convolutional neural network. Dentomaxillofac. Radiol..

[28] Sukegawa S (2022). Identification of osteoporosis using ensemble deep learning model with panoramic radiographs and clinical covariates. Sci. Rep..

[29] Tassoker M, Öziç MÜ, Yuce F (2022). Comparison of five convolutional neural networks for predicting osteoporosis based on mandibular cortical index on panoramic radiographs. Dentomaxillofac. Radiol..

[30] Fukuda M (2020). Comparison of 3 deep learning neural networks for classifying the relationship between the mandibular third molar and the mandibular canal on panoramic radiographs. Oral Surg. Oral Med. Oral Pathol. Oral Radiol..

[31] Vinayahalingam S, Xi T, Bergé S, Maal T, de Jong G (2019). Automated detection of third molars and mandibular nerve by deep learning. Sci. Rep..

[32] Choi E (2022). Artificial intelligence in positioning between mandibular third molar and inferior alveolar nerve on panoramic radiography. Sci. Rep..

[33] Yang S (2022). Development and validation of a visually explainable deep learning model for classification of C-shaped canals of the mandibular second molars in periapical and panoramic dental radiographs. J. Endod..

[34] Jeon S-J (2021). Deep-learning for predicting C-shaped canals in mandibular second molars on panoramic radiographs. Dentomaxillofac. Radiol..

[35] Maia PRL, Tomaz AFG, Maia EFT, Lima KC, Oliveira PTD (2021). Prevalence of soft tissue calcifications in panoramic radiographs of the maxillofacial region of older adults. Gerodontology.

[36] Paju S (2021). Carotid artery calcification in panoramic radiographs associates with oral infections and mortality. Int. Endod. J..

[37] Çetin MB, Sezgin Y, Yilmaz MNN, Seçgin CK (2021). Assessment of carotid artery calcifications on digital panoramic radiographs and their relationship with periodontal condition and cardiovascular risk factors. Int. Dent. J..

[38] Rangarajan K (2021). Artificial intelligence-assisted chest X-ray assessment scheme for COVID-19. Eur. Radiol..

[39] Tam M (2021). Augmenting lung cancer diagnosis on chest radiographs: Positioning artificial intelligence to improve radiologist performance. Clin. Radiol..

[40] Reitsma JB (2005). Bivariate analysis of sensitivity and specificity produces informative summary measures in diagnostic reviews. J. Clin. Epidemiol..

[41] Lee JH, Kim DH, Jeong SN, Choi SH (2018). Detection and diagnosis of dental caries using a deep learning-based convolutional neural network algorithm. J. Dent..

